# Active learning strategies for robotic tactile texture recognition tasks

**DOI:** 10.3389/frobt.2024.1281060

**Published:** 2024-02-06

**Authors:** Shemonto Das, Vinicius Prado da Fonseca, Amilcar Soares

**Affiliations:** ^1^ Department of Computer Science, Memorial University of Newfoundland, St. John’s, NL, Canada; ^2^ Department of Computer Science and Media Technology, Linnaeus University, Växjö, Sweden

**Keywords:** tactile sensing, texture classification, active learning, class imbalancement, time series, temporal features

## Abstract

Accurate texture classification empowers robots to improve their perception and comprehension of the environment, enabling informed decision-making and appropriate responses to diverse materials and surfaces. Still, there are challenges for texture classification regarding the vast amount of time series data generated from robots’ sensors. For instance, robots are anticipated to leverage human feedback during interactions with the environment, particularly in cases of misclassification or uncertainty. With the diversity of objects and textures in daily activities, Active Learning (AL) can be employed to minimize the number of samples the robot needs to request from humans, streamlining the learning process. In the present work, we use AL to select the most informative samples for annotation, thus reducing the human labeling effort required to achieve high performance for classifying textures. We also use a sliding window strategy for extracting features from the sensor’s time series used in our experiments. Our multi-class dataset (e.g., 12 textures) challenges traditional AL strategies since standard techniques cannot control the number of instances per class selected to be labeled. Therefore, we propose a novel class-balancing instance selection algorithm that we integrate with standard AL strategies. Moreover, we evaluate the effect of sliding windows of two-time intervals (3 and 6 s) on our AL Strategies. Finally, we analyze in our experiments the performance of AL strategies, with and without the balancing algorithm, regarding f1-score, and positive effects are observed in terms of performance when using our proposed data pipeline. Our results show that the training data can be reduced to 70% using an AL strategy regardless of the machine learning model and reach, and in many cases, surpass a baseline performance. Finally, exploring the textures with a 6-s window achieves the best performance, and using either Extra Trees produces an average f1-score of 90.21% in the texture classification data set.

## 1 Introduction

Tactile perception in robots refers to their capability to detect and comprehend physical contact, pressure, and vibration information using dedicated sensors integrated into their bodies or end-effectors. Robots can use tactile sensing to augment their perception and interaction capabilities, enabling them to perform tasks with increased precision. Effective tactile perception allows the estimation of crucial information about the surrounding environment in various scenarios, especially under reflection, cluttered environments, challenging light conditions, and occlusion. When relying solely on vision, robots can only identify familiar surface materials and cannot estimate their physical properties independently (Luo et al., 2017). Robots can comprehend and actively engage with their surroundings when receiving static and dynamic sensory information during tactile sensing (Sankar et al., 2021). Tactile perception empowers robots to adapt their joints, links and reactions based on tactile feedback, resulting in improved manipulation, object recognition, and social interactions. Tactile sensing works harmoniously with other sensing modalities like vision and proximity sensing, creating a comprehensive and versatile robot perception system.

Touch is a vital modality while sensing the physical engagement of robots with their environments (Li et al., 2020). Luo et al. (2017) pointed out the importance of utilizing the sense of touch to discern material properties, as it allows robots to determine essential characteristics such as surface texture (friction coefficients and roughness) and compliance, which play a vital role in object manipulation. On the other hand, fine textures need to be slid across a surface to produce micro-vibrations that may then be analyzed and categorized. Luo et al. (2017) emphasized the significance of incorporating the sense of touch to perceive material properties. This capability enables robots to discern essential characteristics like surface texture, including friction coefficients and roughness, and compliance, which are crucial for effective object manipulation. Moreover, tactile-enabled manipulation can improve robot tasks when incorporating exploratory strategies. When analyzing and categorizing fine textures, the robot needs to slide across a surface, producing micro-vibrations that can be analyzed and categorized.

Tactile sensors such as capacitive (Taunyazov et al., 2019; Pagoli et al., 2022), magnetic sensors (Yan et al., 2022; Bhirangi et al., 2021), have been developed for robots to recognize their environment better. In De Oliveira et al. (2015a), the profile of a surface was recognized by dynamic touch using a robotic finger. The motor and inertial measurement unit (IMU) feedback provided was passed through a neural network to classify shapes. The authors of Lederman and Klatzky (1987) pointed out six different exploratory movements to identify the properties of an object. Authors of de Oliveira et al. (2015b) proposed a data-driven analysis for shape discrimination tasks using a robotic finger that performs the sliding movement. Lima et al. (2020) developed an experiment with a sliding tactile-enabled robotic fingertip to explore textures dynamically. The robotic fingertip’s design contains a multimodal tactile sensor that makes contact with the surface. Then, supervised machine learning models were used on this collected data to classify textures. Due to the nature of several textures to have essential features in different directions, this work was further developed in Lima et al. (2021) by doing a two-dimensional exploration of surfaces. Here, the authors also investigated the classification accuracy of machine learning models on tactile data from a multimodal sensing module in a dynamic exploration environment at three different velocities. Von Drigalski et al. (2017) also classified texture on the data collected by a 3-axis force tactile sensor attached to a robot’s gripper. Huang and Wu (2021) classified texture collected by a tactile sensor using a convolutional neural network. Gao et al. (2020) used the BioTac sensor and auto-encoders to improve material Classification Performance. While achieving good results, the sensor used in this work does not measure non-normal forces and changes in exploration direction, which represents a significant difference from the sensor employed in our study. Similarly, the iCub RoboSkin in Xu et al. (2013) incorporates a non-compliant fixture, introducing substantial differences in the data characteristics compared to the information we target using the current dataset. Numerous studies have focused on gathering more detailed tactile data for recognizing texture, resulting in abundant data for performing experiments in tactile sensing. Also, the data in those works are the means of teaching the robots to recognize textures. Thus, a sufficiently labeled dataset is required for texture classification using machine learning models.

The effectiveness of a classification model heavily depends on the data used for training. In Billard and Kragic (2019), the authors state that robotic manipulation using machine learning in unstructured environments is computationally expensive and time-consuming. However, despite the advantages of having abundant data in tactile sensing, the rapidly increasing volume of data also brings specific challenges and drawbacks that require attention. In particular, the scarcity of annotated training data has become a significant challenge for supervised learning techniques since they rely on well-annotated data for effective training. Labeling a large amount of data is costly and time-consuming and often necessitates the expertise of domain specialists (Júnior et al., 2017). To address this challenge, researchers have introduced the concept of Active Learning (AL) (Settles, 2009; Löffler and Mutschler, 2022). AL offers a solution by enabling the model to actively select and acquire the most informative data points for annotation, thus reducing the need for large amounts of labeled data. The core concept of AL is that if the learning algorithm can choose the data from which it learns, it will perform better with less training data (Settles, 2009). In scenarios where the strategy asks an expert for labels, different kinds of query strategies pave the way for deciding which instances are the most informative to be labeled. There have been many proposed ways of formulating such query strategies in the literature (Settles, 2009). Among the query strategies, Uncertainty (UNC) sampling is the simplest and most commonly used (Lewis and Gale, 1994). The AL system queries the expert to label the most informative instance (i.e., the instance(s) that a machine learning model is most uncertain about its class) in UNC. There are numerous metrics to calculate uncertainty, some of which are Least Confident (Settles and Craven, 2008), Margin Sampling (Settles, 2009), and Entropy (Shannon, 1948). Another popular query strategy is Query By Committee (QBC) (Settles, 2009). This AL strategy selects the most informative data points for labeling by querying regions in the input space where the models in a committee disagree. Moreover, to implement QBC, a measure of disagreement among the committee members must be established to identify the data points where the models disagree and are uncertain (Settles, 2009). Finally, the Expected Model Change (EMC) (Freytag et al., 2014) strategy, which predicts the influence of an unlabeled example on future model decisions and if the unlabeled example is likely to change future decisions of the model when being labeled, it is regarded as an informative sample.

Over the last decade, studies have started incorporating AL strategies into robotics, realizing the necessity and importance of well-annotated data to teach the robots better. In Stanton (2018), a robot conducts unsupervised discovery to get the data it needs in dynamic settings where labeled datasets are absent. The authors of Taylor et al. (2021) termed AL a process in which agents make decisions to collect the most relevant data to achieve the desired learning objective. They also pointed out that usually informative samples are sparse and believed AL could fetch those samples to the robots for training, thus reducing the labeling cost and time. Chao et al. (2010) discussed that AL is a transparent approach to machine learning as the algorithm queries an expert that provides information about areas of uncertainty in the underlying model. In their research, they implemented AL on the Simon robot and found potential improvement in the learning process’s accuracy and efficiency. AL has also been used for robotic grasping. For instance, in Wei et al. (2023), authors developed a Discriminative Active Learning (DAL) framework, evaluated real-world grasping datasets, and performed better with less annotated data. Moreover, they showed model trained with fewer data selected by this AL framework could handle the task of real-world grasp detection. Another framework was suggested for recognizing objects and concept acquisition in Gkatzia and Belvedere (2021). This framework’s combination of few-shot learning and AL reduced the need for robot data annotation. In their study, (Sheikh et al., 2020) addressed AL in the context of semantic segmentation to reduce the human labeling effort of image data obtained from a mobile robot. Their strategies resulted in achieving higher accuracy with a reduced number of samples. To enhance object detection with limited annotated data, the authors of Xu et al. (2020) focused on exploiting canonical views through an active sampling approach named OLIVE. This method selects optimal viewpoints for learning using a goodness-of-view (GOV) metric, combining model-based object detection consistency and informativeness of canonical visual features. Samples chosen by OLIVE, along with data augmentation, are used to train a faster R-CNN for object detection. It is crucial to underscore that including data augmentation in their pipeline introduces an additional processing cost. While such a strategy effectively alleviates the burden of labeling data, it necessitates higher-cost robotic hardware for optimal performance. Their study validates the principle of active sampling in the context of robot learning for object detection, and it aligns with our goal of minimizing annotated data required for robot training. In contrast to OLIVE, which uses the GOV metric, we utilize traditional AL strategies, namely, UNC, QBC, and EMC, along with their uncertainty metrics to identify informative samples. This reduces the labeled data requirement and, like OLIVE, enhances training efficiency, improving texture recognition performance. Notably, the additional processing entailed by data augmentation in OLIVE is not present in our pipeline, rendering our strategy better suited for scenarios where lower-cost robotic hardware and less complex models are preferred.

Our work focuses on the potential significance of Active Learning (AL) in tactile sensing for texture recognition, particularly in the context of future robotic manipulation in unstructured environments. Recent studies (Cui and Trinkle, 2021; Ravichandar et al., 2020) highlight the significance of learning from demonstration (LfD) as the paradigm in which robots acquire new skills by imitating an expert. In addition, labeling objects from daily activities with the help of a human specialist will become fundamental for integrating robotic manipulation in unstructured environments, such as homes, universities, hotels, and hospitals. In this work, we have utilized AL to enhance the texture classification process by considerably reducing the training size of machine learning models.

Additionally, the data obtained from tactile sensors consists of time series, resulting in a large dataset to be processed. The volume of the collected data increases with the frequency at which it is collected, making tactile data more complex and increasing the labeling cost. It is worth mentioning that there have been previous studies where AL has been successfully applied to time series data. In Wu and Ortiz (2021), AL has performed better using fewer samples on real and synthetic time series datasets. Authors in He et al. (2015) used AL to get adequate, reliable, annotated training data for multivariate time series classification. Often, time series data have a very imbalanced data distribution. This may result in bias in the AL strategies while selecting the most informative data points and, thus, in the whole training process. He et al. (2015) have addressed the need to balance the training data among different classes while performing the classification task and believe this affects the model’s generalization. Moreover, while analyzing or classifying time series, temporal features play an essential role. Wang et al. (2014) have pointed out how rarely the time variable’s effect is considered. Their study shows that by using more temporal information, the partitioning method results in greater forecasting accuracy for sensor inputs at the feature extraction stage, and the data should be segmented into smaller segments at the feature extraction stage for sensor inputs. For the recognition task, the authors in Ma et al. (2020) mentioned that when the window size is too short, some actions may be split into numerous consecutive windows, activating the recognition task too frequently without producing high recognition results. According to Jaén-Vargas et al. (2022), it is not always necessary to have large sliding windows to achieve higher performance. In the current paper, we aimed for a tradeoff between the information required for recognition and the cost of processing.

This study investigates the effects of using AL strategies for texture classification using tactile sensing. We envision a future where automated systems operate in uncertain and unstructured environments, lacking access to reliable analytic models or extensive historical datasets. In such scenarios, active learning and data-driven control could become paramount. In practical scenarios, a robot capable of obtaining labels from a specialist could apply active learning strategies to select the most informative samples and obtain precise information to update its world model. However, without such a strategy, the sheer volume of data queries might render it unfeasible for a human specialist to assist the robotic platform effectively.

Due to the multi-class classification (12 classes) nature of our problem, we propose a class-balancing instance selection algorithm to address the imbalance issues that may arise from standard AL strategies when selecting instances for labeling. This algorithm is integrated into standard AL strategies (UNC, QBC and EMC) to improve the performance of texture classification models, allowing them to achieve competitive or superior performance with fewer training instances when compared to a baseline using the entire dataset. Additionally, we use a sliding window approach to extract features from time-series data. We compare the effects of temporal features using two different window sizes, aiming to reduce further the training data size for robots’ texture classification tasks. In summary, our proposed strategy combines a sliding window strategy, time-series feature extraction, and active learning with a class-balancing instance selection algorithm to reduce the number of training instances for classifying textures with supervised learning models. Our research significantly contributes to tactile texture recognition and robotic exploration in unstructured environments. We introduce a pipeline for data pre-processing and novel class-balancing technique within the context of active learning, addressing the challenge of imbalanced datasets in tactile texture classification. Our approach enhances classification performance and demonstrates the potential for robots to adapt and learn efficiently from limited human supervision, paving the way for future autonomous robotic manipulation in diverse and uncertain real-world settings.

## 2 Materials and methods

**FIGURE 1:**
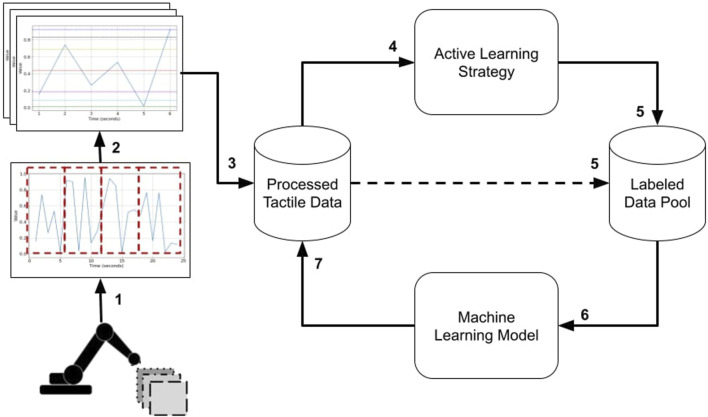
The pipeline for tactile data used for texture classification. 1) Data collection from exploratory movements; 2) time series data is partitioned into temporal; 3) statistical attributes extraction; 4) using AL strategies to rank instances; 5) the AL strategy selects top-ranked instances; Machine-learning model built with the instances in the labeled pool; 7) classify all instances in the processed tactile data pool.

The entire pipeline developed in this work is presented in Figure 1. First, in step 1, the data used in this work was collected through exploratory movements of a tactile sensor-equipped robotics finger. The data is available in (Monteiro Rocha Lima et al., 2023; Lima et al., 2023), and the experimental setup is explained in Section 2.1. The time series data is subsequently partitioned into more manageable temporal windows in step 2. Extracting an array of statistical attributes from these shorter windows generates the features and processed tactile data for our machine-learning models in step 3. After, we use AL strategies to rank instances based on how informative they are for requesting labels (step 4), and the top-ranked instances are selected by the AL strategy (step 5). Subsequently, these instances are attributed their appropriate labels, as already encoded in the processed data, and are consequently included in the annotated data pool (step 5). Then, we build a machine-learning model with the instances in the labeled pool (step 6) and classify all instances in the processed tactile data pool (step 7). As the pipeline unfolds iteratively, steps 4 through 7 are recurrently executed until a predefined upper limit of instances is achieved, which is constrained by a pre-established budget (i.e., a maximal budget). Ultimately, our approach’s outcome is a machine learning model trained by an AL strategy and a maximal budget smaller than the total number of instances available in the original dataset. All essential processes described in Figure 1 of the entire pipeline are detailed in the following subsections.

### 2.1 Experimental setup and texture data collection

**FIGURE 2:**
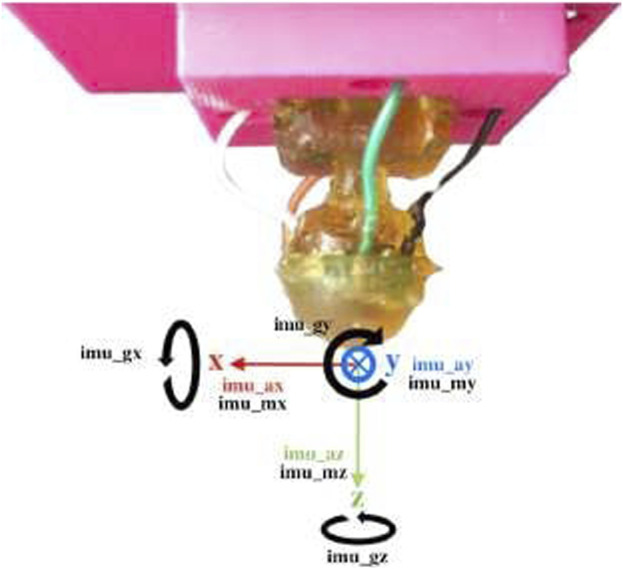
Multi-modal bio-inspired sensor and MARG frames of reference (Lima et al., 2021).

In this study, we used the tactile data available in (Monteiro Rocha Lima et al., 2023; Lima et al., 2023), which was collected from a robotics finger from a previous study by Lima et al. (2021) and represents step 1 in Figure 1. The fingertip of the robotic finger was equipped with a fixed miniaturized tactile sensor, which was developed by De Oliveira et al. (2017). This miniaturized sensor was also used in a previous study by Lima et al. (2020). The scaled-down version of this module is depicted in Figure 2.

**FIGURE 3:**
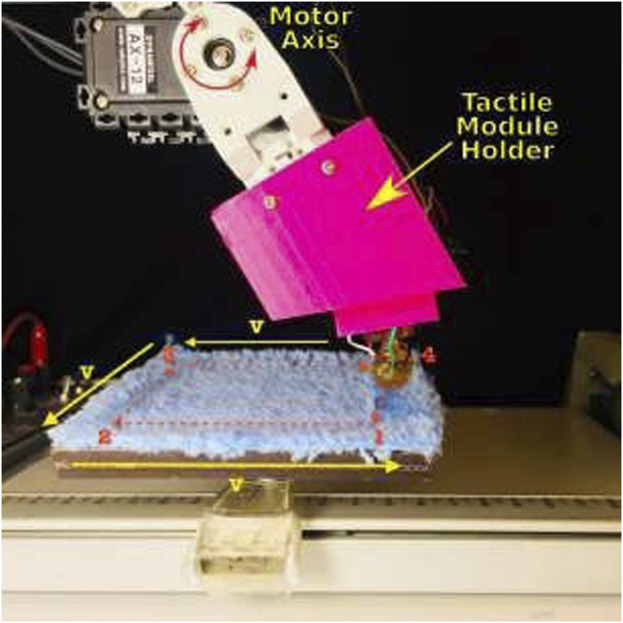
XY-recorder setup with a texture under exploration (Lima et al., 2021).

Different from (Lima et al., 2020), the tactile sensing module used in this study has a rounded profile and flexible materials that enable exploratory motions, resembling the shape of a human fingertip. During experiments, the sensor was securely held in place by an articulated robotic finger mounted on a plastic base. The rounded form of the fingertip aims for a more straightforward exploration of 2D textures. The module incorporates a Magnetic, Angular Rate, and Gravity (MARG) system with nine degrees of freedom, providing valuable information about the exploration vibrations. Additionally, the module features a base-mounted barometer that captures deep-pressure data. Even though this unique sensor setup also allows us to record the module’s deformation, in the present work, we focus on classifying textures based on the pressure data from the barometer. Figure 3 shows the sensing module holder intended to mimic the index finger’s natural probing. The motor housing serves as a permanent representation of the intermediate phalange. The motor axis is used to represent the intermediate-distal joint, and the tactile module holder is used to describe the distal phalange. The distal phalange moves to bring the sensor module into touch with the surface as the intermediate-distal joint rotates.

**FIGURE 4:**
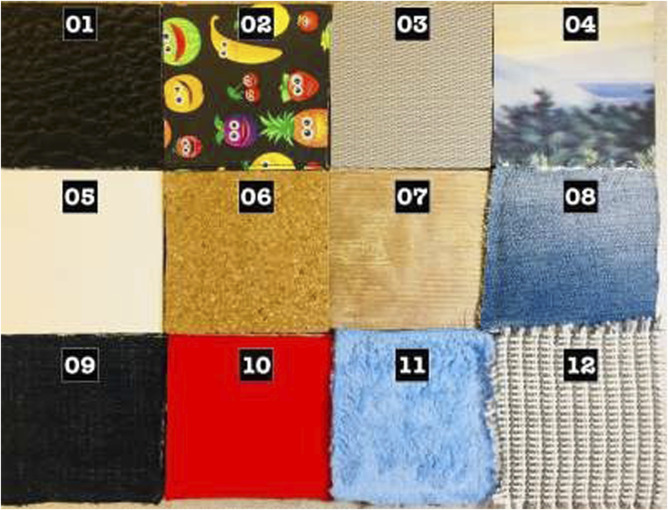
The set of textures explored in the dataset (Lima et al., 2021).

The data (Monteiro Rocha Lima et al., 2023; Lima et al., 2023) used here contain readings of 12 textures as shown in Figure 4. More details regarding the textures are available in Lima et al. (2021). The surface containing the textures was explored in a square orientation (i.e., exploring *X* and *Y* directions), as depicted in Figure 3. Each experimental trial involved completing a square for each texture, with 100 experimental runs conducted at three different velocities (30, 35, and 40 mm/s). The dynamic exploration began by lowering the fingertip until it made contact with the textured surface. A small torque was applied to the fingertip to ensure consistent contact throughout the experiment. An empirically chosen pressure threshold determined when the fingertip torque would end. The total run time for each experiment was 12 s, with 3 s required to travel each side of the square. The dataset uses a unique compliant multi-modal tactile sensor comprising a deep pressure sensor and inertial measurements, which provides the interesting ability to detect micro-vibrations and changes in pressure non-normal to the surface while changing directions, as performed in this dataset, which is expected from robots exploring daily textures. More details regarding the experimental setup and data collection approach can be found in Lima et al. (2021).

### 2.2 Data preprocessing and feature extraction

Based on the findings of Lima et al. (2021), the barometer feature achieved the best classification results, exhibiting 100% accuracy for most texture classification cases. Therefore, we decided to rely solely on the barometer sensor to conduct our experiments to investigate dimensionality reduction. For our work, we utilized 1,200 samples (corresponding to 12 textures) collected solely using the barometer at a velocity of 30 mm/s. Each sample contains data collected at 350 Hz while exploring the respective textures. Subsequently, we applied our preprocessing pipeline to these raw samples. The pipeline involves using an overlapping sliding window with a configurable window size, where the overlap was set to 50% of the window size. We believe setting higher overlapping thresholds could result in excessively similar examples within the sequence, potentially reducing diversity in the training set and increasing the processing time required to transform readings into machine-learning-ready examples. The consequence of such higher overlapping thresholds is that it could pose challenges in resource-constrained environments. We experimented with two window sizes (3 and 6 s) to explore the impact of temporal features on our texture classification task. The values of 3 and 6 s used in this work were set mainly because of the setup of how the data was collected. A sliding window of 3 s corresponds to 25% exploration, while 6 s corresponds to 50% exploration. In the experiments, we aim to capture distinct dimensions of exploration within these time slices, and extending the exploration beyond 6 s would lead the module to revisit dimensions already explored in the initial 3 s, resulting in redundant data collection For each window, the pipeline generates a scaled instance (i.e., a MinMax scaler with values ranging from 0 to 1) comprising 11 statistical features, including mean, median, variance, skewness, standard deviation, quantiles (10, 25, 76, 90), min, and max. In this way, a resultant statistical features data frame is generated for each time window on which the machine learning models are trained.

**FIGURE 5:**
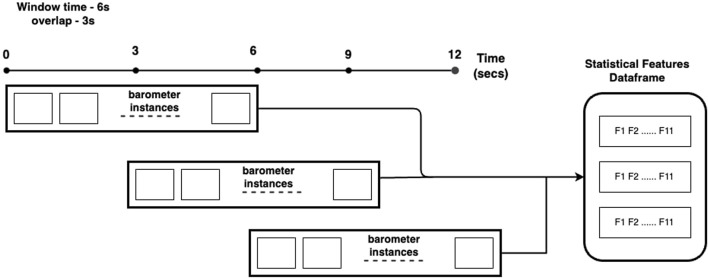
Window-based statistical features extraction for the 6 s with 3 s overlap.

This generation of features uses the window strategy depicted in Figure 5 and described below. Given as input a time window of 6 s and an overlap of 3 s, the first instance *i*_1_ generated from one experiment would start on time *t* = 0 and end time *t* = 6. From all the barometer data collected from this time window, we extract the 11 statistical features and add such features to a data frame, storing the texture label of that particular instance. For the second instance of the same experiment with a texture, we move the window by 3 s (i.e., start on time *t* = 3 and end time *t* = 9) and repeat the process of extracting features and adding a label to the texture. This entire procedure is repeated until the end of each experiment with the textures. Such an approach generated two datasets, one for a 3 s window with 50% overlap consisting of 6,718 instances, and another for a 6 s window with 50% overlap and 2,878 instances. The processed data (step 3) referred to in this subsection is the processed tactile data discussed in Figure 1.

### 2.3 Active learning strategies and class-balancement instance selection algorithm

AL strategies aim to select the most uncertain instances classified by a model to be labeled by an expert so that the model can learn better with less data. Our research compares three standard AL approaches: Uncertainty sampling (UNC) (Settles, 2009), Query by Committee (QBC) with bagging (Settles, 2009), and Expected Model Change (EMC). The UNC strategy selects for labeling the most informative instances from an unlabeled data pool in an iterative manner. It measures the model’s uncertainty about its predictions using metrics such as entropy, margin sampling, or least confident predictions. The labeled data is then used to improve the model through training updates. QBC with Bagging is an active learning strategy combining QBC with a bagging ensemble. It generates diverse models using bootstrapped sampling and identifies uncertain instances by assessing disagreement among the committee of models. These instances with a high degree of uncertainty are labeled, and the ensemble is continually updated with the new labeled data. The EMC strategy identifies instances expected to induce the maximum positive change in the model’s performance when labeled. This approach estimates the potential improvement by evaluating the impact of each unlabeled instance on the model’s behavior, and the instance with the highest expected positive change is selected for labeling.

In our experiments, we split 80% of our processed data as a training set and 20% into a test set for validation purposes. In our AL strategy, the training set is used as an unlabelled pool (i.e., the Processed Tactile Data in Figure 1) from which the most uncertain labeled instances are queried for being labeled. The labels are extracted from the raw data obtained by the robotic arm with tactile sensors, and therefore, we do not involve a user annotating the data in our experiments. Once an instance is labeled, it is added to the pool of labeled data on which a machine-learning model can be trained. As AL is an iterative process, we fix a maximum annotation budget (i.e., a maximum number of instances that can be queried throughout the process) and a step size that defines the number of instances to be labeled at each batched query we make on the unlabeled pool. The whole experiment was controlled and repeated 20 times. We used 20 seed values for experimental reproducibility. The seed generation procedure used the decimals of *π* selected four by four (i.e., *seed*_1_ = 1415, *seed*_2_ = 9265, etc.). Such a decision avoids an arbitrary choice of seeds. At the first iteration of the AL strategies, instances are queried randomly (step 4 in Figure 1). After we obtain the first subset of labeled training data, and then the AL strategy is executed (step 5 in Figure 1), ranking and selecting the most informative instances to be labeled. For UNC, we used Least Confidence (Settles and Craven, 2008) as a metric for evaluating the uncertainty of instances defined in Eq. 1. Using Eq. 1 will query the instances with the lowest confidence and the highest uncertainty for getting labeled. Here, y* is the most likely class label assigned by a machine learning model.

$$\phi^{\text{LC}}\left(x\right)=1-P\left(y^{*}|x;\theta \right)$$ (1)

While implementing QBC with bagging, we have created a committee with base models trained on different subsets of labeled data using bagging. The committee of models collectively predicts the labels for unlabeled instances or candidate samples. Each model in the committee provides its prediction for each instance, and the level of disagreement among the committee members is measured to assess the uncertainty or informativeness of each candidate sample. For our case, we have used vote entropy, shown in Eq. (2), to calculate the disagreement among the committee members. Using Eq. (2), the most informative samples were queried from the unlabelled pool and added to the labeled pool for training. In Equation (2), *V* (*y*_*t*,*m*_) is the number of votes a particular label m receives from the committee of classifiers.

$$\phi^{VE}\left(x\right)=-\frac{1}{T}\overset{T}{\underset{t=1}{\sum }}\overset{N}{\underset{n=1}{\sum }}\frac{V\left(y_{t,n}\right)}{C}\frac{\log V\left(y_{t,n}\right)}{C}$$ (2)

We instantiated the Expected Model Change (EMC) through Eq. (3), which quantifies the performance differential resulting from incorporating an unlabeled instance. This instance is assigned a potential label that maximizes the expected impact on performance. In Equation (3), *x* is the instance to be evaluated, *y*_*i*_ are all labels possible in a given data set, and *M* is a performance metric. This work used the f1-score as our performance metric to calculate the EMC.

$$\phi^{MC}\left(x\right)=\underset{\left\{y_{i}\in \left[l_{1},l_{k}\right]\right\}}{\max}\left(M\left(x,y_{i}\right)-M\right)$$ (3)

This paper presents a novel class-balancing instance selection algorithm that effectively addresses the class imbalance issue in the data. The main objective is to query the unlabeled instance pool in a manner that ensures a balanced representation of classes in the labeled data. This approach aims to prevent bias in the model’s classification performance, which may occur when certain classes have a disproportionate number of instances selected by an active learning (AL) strategy. The core idea behind our algorithm is to modify the class frequencies in the training set by inverting their occurrences. Consequently, we prioritize selecting more instances from classes with fewer instances in the training set while reducing the number of instances chosen from classes with more instances. To illustrate, consider a problem with four classes, where the instance frequencies in the training set from an AL strategy are *f*_*class*_1_ = 0.4, *f*_*class*_2_ = 0.15, *f*_*class*_3_ = 0.2, and *f*_*class*_4_ = 0.25. In the next round of instance selection to be labeled by an expert, we would invert these frequencies, leading to the selection of 15% of instances from class 1, 40% from class 2, 25% from class 3, and 20% from class 4. By employing this strategy, we endeavor to achieve a balanced training set. However, it is essential to acknowledge that perfect balance cannot be guaranteed, given uncertainties associated with the class label of the selected instance, which the expert will ultimately resolve. In summary, our proposed class-balancing instance selection algorithm offers a promising approach to address a class imbalance in active learning, mitigating potential bias and enhancing the overall classification performance by striving for a more equitable representation of classes in the training data.

A pseudo-code of our strategy is given in Algorithm 1. In summary, the method selectively adjusts the class frequencies to emphasize classes with lower representation while reducing the prevalence of classes with higher representation. This process enhances the balance in class distribution, which is crucial for the better performance of various machine-learning algorithms. In line 1, the algorithm starts by computing the frequency of each class in the training data. The *bincount* () function is used to count the occurrences of unique labels in *current*_*train*_*y*, resulting in an array named *freq*. In line 2, the frequency values obtained in the first step are normalized to a range between 0 and 1. Each frequency value is divided by the number of examples in the training set (len (*current*_*train*_*y*)) to yield a new array named *norm*_*freq*. To achieve an inverted probability distribution, the *norm*_*freq* array is sorted in descending order using the *sort* () function, and the [:: − 1] slicing is applied to reverse the order of the sorted array. The resulting array is named *sorted*_*freq* (line 3). Next, to determine the mapping that would invert the probabilities, an auxiliary array named *sorted*_*indices* is created (line 4). This array stores the indices that would sort the *norm*_*freq* array in ascending order when sorted twice using the *argsort* () function. Then, a loop (lines 5–7) is executed, iterating over each element in *sorted*_*indices*. For each index *i*, the corresponding value in *sorted*_*freq* is assigned to the *inverted*_*freq* list at the position *i*. After all iterations, the algorithm has constructed the *inverted*_*freq* list, which now contains the inverted frequency values for each class in the training set that is returned as the result of the algorithm in line 8. This algorithm was integrated with both AL strategies, and its impact on AL strategies is presented in the Results section.

Algorithm 1 Compute Inverted Example Frequency

* *
**Input:** *current*_*train*_*y*: all label values used in the current training set
* *
**Output:** *inverted*_*freq*: The inverted frequency of the class distribution
1:** *freq* ←bincount (*current*_*train*_*y*)
2:** $\mathrm{norm\_freq}\leftarrow \frac{\mathrm{freq}}{\text{len}(\mathrm{current\_train\_y})}$

3:** *sorted*_*freq* ←sort(*norm*_*freq*)[:: − 1]
4:** *sorted*_*indices* ←argsort(argsort(*norm*_*freq*))
5:** **for** *i* **in** *sorted*_*indices* **do**
6:* *  *inverted*_*freq*[*i*] ← *sorted*_*freq*[*i*]
7:** **end** **for**
8:** **return** *inverted*_*freq*

### 2.4 Classification models and evaluation metric

In our active learning approach, we can integrate any classifier as a model to predict the instance classes (step 6 in Figure 1). For our experiments, we opted for widely-used classifiers available in the *sklearn* package, including Decision Trees (Rokach and Maimon, 2005), Extra Trees (Geurts et al., 2006), XGBoost (Chen and Guestrin, 2016), and Random Forest (Breiman, 2001). A decision tree recursively splits the dataset into subsets based on the most significant feature at each tree level, making decisions until it reaches a decision at the leaf nodes. A Random Forest is an ensemble model that collects weak learners’ predictions and aggregates their individual predictions to produce a final and more robust forecast. The Extra Trees (i.e., Extremely Randomized Trees) model is also an ensemble algorithm that builds multiple decision trees, but it introduces additional randomness on the section of the feature and thresholds in the tree-building process. Finally, the XGBoost (i.e., Extreme Gradient Boosting) is an ensemble model trained sequentially and uses the gradient descent optimization technique to minimize a loss function while adding weak learners to the ensemble. Throughout our experiments, we used the base version of these classifiers from the scikit-learn package (Pedregosa et al., 2011), employing their standard hyperparameters detailed in Table 1. All ensemble techniques (i.e., Random Forest, Extra Trees, XGBoost) used a Decision Tree as the weak learner.

**TABLE 1:** Models’ hyperparameter settings.

Model	Hyperparameters
Decision Tree	*split criterion:* gini index
	*minimal samples to split:* 2
Extra Trees	*split criterion:* gini index
	*minimal samples to split:* 2
	*number of estimators:* 100
Random Forest	*split criterion:* gini index
	*minimal samples to split:* 2
	*number of estimators:* 100
XGBoost	*learning rate:* 0.3
	*max depth:* 6
	*number of estimators:* 100

This choice ensures the classifiers are utilized in their default configurations, allowing for fair and consistent comparisons during our evaluations. By employing these standard classifiers, we aim to comprehensively assess their performance in the context of active learning for texture classification tasks. In this work, we chose to use shallow models because one of the primary considerations is that processing efficiency is paramount in the context of embedded systems. Therefore, using deep models can introduce significant computational burdens that may not align with the expected processing capabilities of robotic platforms operating in unstructured environments.

Our work adopted a different evaluation metric than the approach used in Lima et al. (2021). Instead of using accuracy, we utilized the f1-score as our evaluation metric. The choice of f1-score as the evaluation metric is supported by Sokolova and Lapalme (2009), where it was highlighted that accuracy, although commonly used for classification performance evaluation, may not adequately address class imbalance issues. While our data may be balanced, our active learning (AL) strategies could still suffer from the effects of class imbalance while selecting instances from different classes in its iterative process. Hence, we deemed it necessary to consider the f1-score as our evaluation metric. Unlike accuracy, the f1-score considers precision and recall, making it a more balanced and informative evaluation metric, especially in scenarios involving imbalanced class distributions. By incorporating both precision and recall, the f1-score provides a comprehensive assessment of the classifier’s performance, ensuring a more robust evaluation of our texture classification task. The models produced at this step are iteratively trained in the labeled data pool. At every training cycle (steps 4 to 7 in Figure 1), the number of labeled data inside the pool is increased and we record the model’s achieved performances for an in-depth evaluation of our entire proposed pipeline.

## 3 Results

In this section, we conduct a comprehensive analysis of the performance of our AL strategies using four different classifiers, employing f1-score as the evaluation metric. To establish the baseline performance of each classifier considered in this work, we train and test them using repeated (4 times) 5-fold cross-validation. The baseline f1-score values are a reference for comparing all combinations of machine learning models and AL strategies used in this work since they represent an average performance of the models using the entire dataset for building a model. It is essential to point out that the baseline value is not a ceiling value for the problem discussed in this paper since changing the training data will likely change the decision surface of a machine learning model and, as a consequence, there is a possibility of obtaining a better model than simply using more data to train a model. In this section, we start by exploring the influence of temporal features in the texture classification task by comparing the results obtained using different window sizes (e.g., 3 and 6 s) and window overlaps (e.g., 75%, 50%, 25%). Furthermore, we investigate the impact of our class-balancing instance selection algorithm on the AL strategies by generating results both with and without such an algorithm. This comparison allows us to assess the effect of the class-balancing approach on the performance of the AL strategies. The Figures in this section present the number of instances queried for labeling at each Step (*x*-axis) against the evaluation metric f1-score values (*y*-axis). For each step in the querying process, we present two box plots. The blue box plot shows the performance of the AL strategy (e.g., UNC or QBC) with the class-balancing algorithm, while the red box plot presents the performance without the algorithm. We apply a sliding overlapping window with several durations and overlaps on the raw data to prepare the data for analysis. This preprocessing pipeline generates processed datasets of statistical features, as discussed in Section 2.2. The AL strategies are then executed on these processed datasets, allowing us to examine their effectiveness in texture classification.

### 3.1 Sliding window size and overlap percentage analysis

We start by assessing the influence of the size and duration of the sliding window. We have tested several combinations of parameter values to understand how these values affect our experiments. The sliding window size was tested for 1, 3, 6, and 9 s, as these values are sufficient to test robots exploring the axis of objects in one (e.g., 1 and 3 s) or two directions (6 and 9 s). We also tested overlaps of 75%, 50%, and 25%, aiming at generating more or less data for the machine learning models and evaluating how their performances would be affected by those different amounts of training data. We tested all combinations of these parameter values (4 sliding window sizes and three overlapping values). A sliding window of 9 s with an overlap of 25% cannot be obtained in our experiments since the robot exploration is of at most 12 s, and a 25% overlap would start the next window with 6.75 s and therefore could not obtain a total window size of 9 s (i.e., starting at 6.75 s and ending at 15.75 s). Therefore, we test 11 combinations of sliding window sizes and overlaps. The details of the datasets generated by all these 11 combinations can be seen in Table 2. Finally, we have used a repeated (four times) 5-fold cross-validation for all the machine learning models (e.g., Decision Tree, Extra Trees, Random Forest, and XGBoost), and the average f1-scores are reported in Table 3.

**TABLE 2:** The total number of instances generated by our sliding window procedure when using the 11 sliding window size and overlap combinations.

Sliding window size	Overlapping percentage		
	75%	50%	25%
1 s	53,997	27,598	17,999
3 s	15,599	8,399	5,999
6 s	5,999	3,599	2,400
9 s	2,400	1,200	-

**TABLE 3:** Average f1-score values for all 11 combinations of sliding window sizes and overlaps.

Window sizeOverlap	1 s			3 s			6 s			9 s		
	75%	50%	25%	75%	50%	25%	75%	50%	25%	75%	50%	
Decision Tree	51.21	51.62	55.64	70.78	72.82	75.33	80.90	80.90	79.43	87.82	88.55	
Extra Trees	64.73	63.66	67.04	82.99	83.89	84.63	90.64	89.75	88.36	94.40	93.80	
Random Forest	64.24	63.66	66.80	82.31	83.18	83.92	89.51	88.83	87.54	92.81	91.03	
XGBoost	65.50	64.87	67.41	83.50	84.01	84.29	90.56	89.45	87.56	93.60	92.63	

The results presented in Table 3 show that a window of 1 s is insufficient to distinguish the textures. This means that using a window of 1 s cannot extract meaningful features to classify the textures since the f1-scores are in a range between 51% and 67%. Higher performances are achieved when the robot explores the texture for 3–9 s. It is essential to point out that the objective of our work is to learn with as little data as possible, and we relied on Table 2 to decide on the proper values for the window size and overlap. We discarded 9-s windows mainly because the number of instances to be chosen for an AL strategy would be low (i.e., 1,200 to 2,400 instances) compared with other setups, which could decrease the advantage of selecting high-yield instances from the unlabeled pool. In addition, a robot should explore the surface as little as possible to predict the proper label for the texture and using 9 s would make the robot explore a direction (i.e., x or *y*-axis) a second time. It is important to highlight that higher performance values with longer windows are expected since the robot would explore them for a longer time on the surface and have a higher chance of correctly classifying the texture. Finally, we discarded the overlapping windows of 25% and 75% since the differences between them, when compared to a 50% window, were not statistically significant for 3 and 6 s windows. Therefore, we decided to provide a balance between performance and a reasonable pool of instances to be given to the AL strategies, which is obtained by using the 50% overlap. As a result of this experiment, we conclude that using windows of 3 and 6 s with an overlap of 50% is a reasonable choice (i.e., obtaining higher performances while maintaining a reasonable pool of instances for the AL strategies) of parameters for the data set used in our experiments and, therefore, we use this result to proceed with our AL strategies analysis.

### 3.2 Uncertainty sampling strategy on 3 and 6 s windows

**FIGURE 6:**
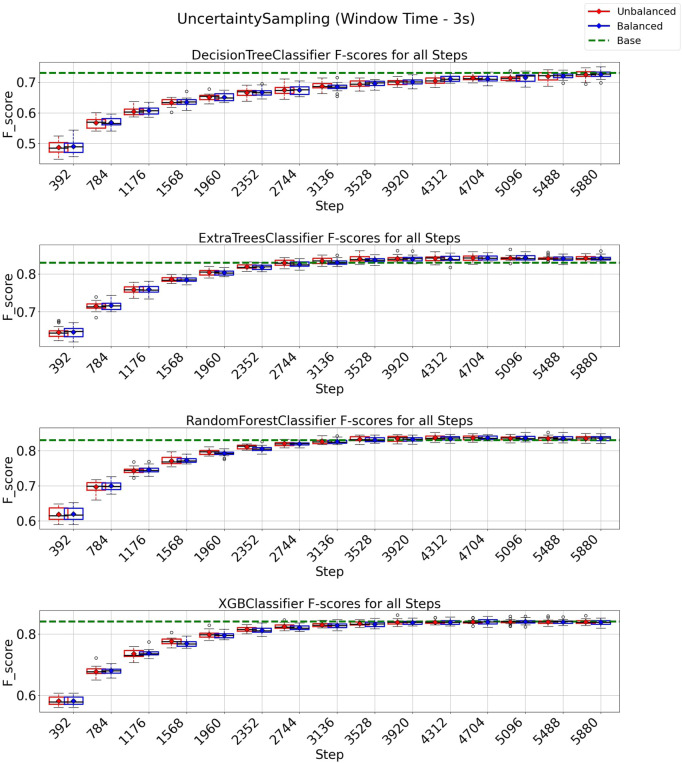
F1-score plots of the uncertainty sampling strategy for the 3-s window.

In this experiment, we adopted the uncertainty sampling (UNC) strategy to minimize the number of instances requiring expert annotation and to enhance texture classification performance. Figure 6 illustrates the results obtained using a window size of 3 s and an overlap of 50%. All classifiers using the UNC strategy managed to achieve and surpass their respective baseline f1-score values within an annotation budget of approximately 5880 out of a total of 8,399 instances (equivalent to 70% of the data). This finding suggests that we could reduce the training set for these classifiers by 30% while achieving comparable or even improved performance compared to utilizing the entire dataset for training. Notably, for the ExtraTrees, Random Forest, and XGBoost classifiers, the total budget could be reduced to only 3,920 instances (equivalent to 47% of the data) to achieve baseline performance. It is also noticeable that UNC with the class-balancement algorithm performed similarly to the standard UNC algorithm, and therefore, no substantial gains were observed by using our class-balance instance selection algorithm.

**FIGURE 7:**
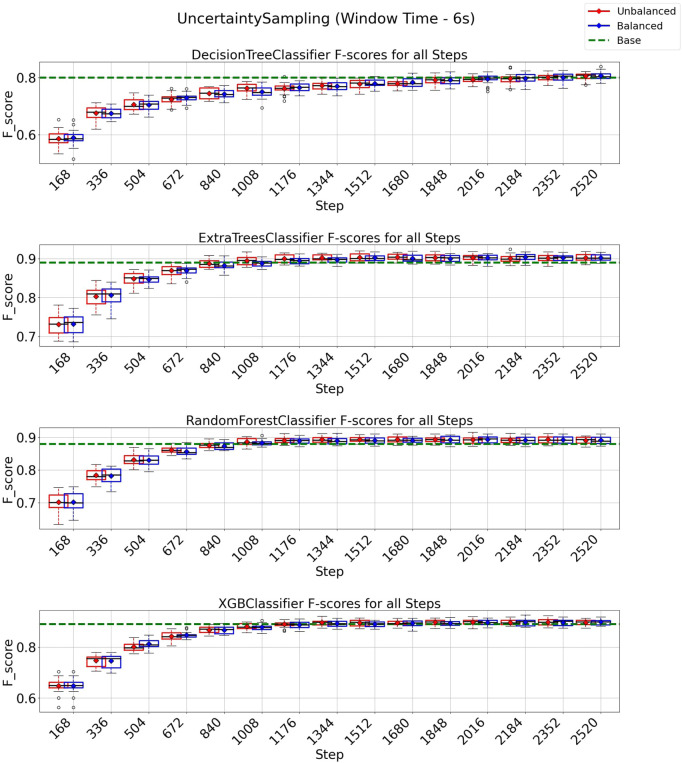
F1-score plots of the uncertainty sampling strategy for the 6-s window.

In Figure 7, we present the results obtained using the uncertainty sampling (UNC) strategy with a window size of 6 s. For this configuration, all classification models achieved performance exceeding the baseline with an annotation budget of 2016 instances (equivalent to 56% of the total 3,599 instances in the dataset). This finding indicates that a substantial reduction of 44% in instances is possible when employing the UNC strategy to train machine learning models in this texture classification task without compromising performance. Again, in the case of Extra Trees, Random Forest, and XGBoost a further reduction for 1,176 instances (33% of all training data) is enough to surpass the baseline, indicating a reduction of 67% of the training data is possible and still achieve the baseline value. Furthermore, when combined with the class-balancing instance selection algorithm, we observed that the UNC strategy demonstrated performance similar to the standard UNC algorithm. This suggests that no significant improvements were achieved by balancing the selection of the next round of instances when using UNC as the active learning strategy.

### 3.3 QBC strategy on 3 and 6 s windows

**FIGURE 8:**
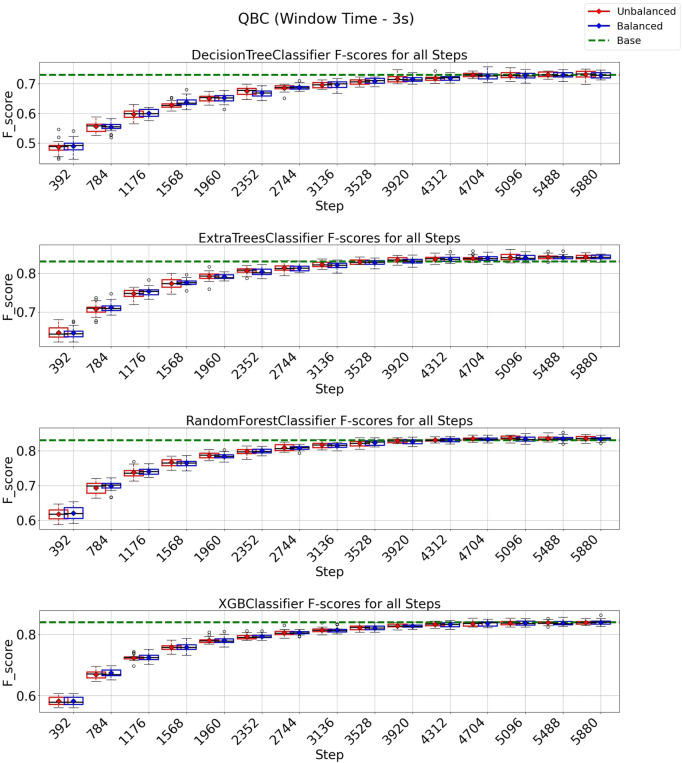
F1-score plots of the query by committee strategy for the 3-s window.

**FIGURE 9:**
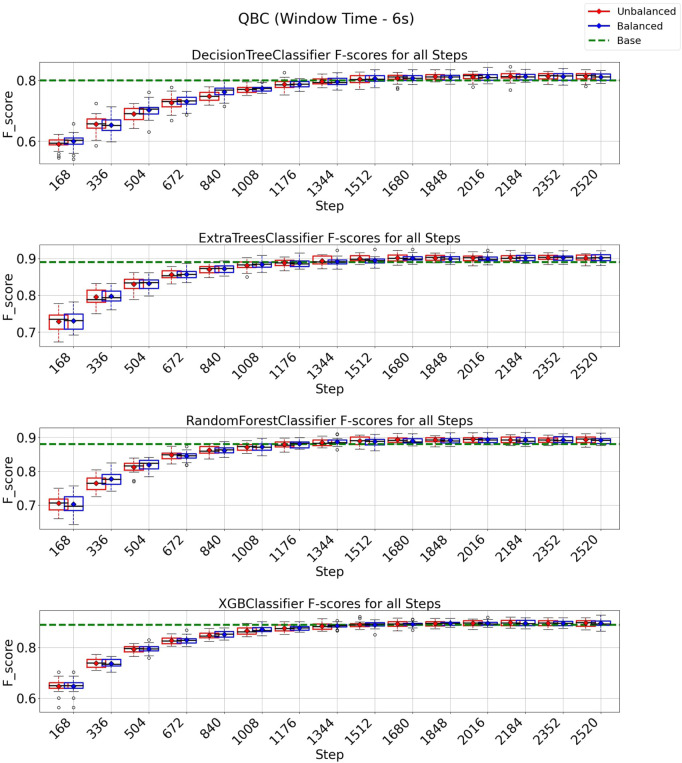
F1-score plots of the query by committee strategy for the 6-s window.

In the case of the QBC strategy, all classification models reach at least their baseline score for a window of 3 s, as shown in Figure 8 with a budget of 5880 instances from a total of 8,399 instances (70% of the entire data set). It is essential to observe from the experiments depicted in Figure 8 that the class-balancing algorithm indeed increased the performance of the QBC strategy compared to the standard QBC implementation, mainly in the early cycles of the QBC strategy. For a window of 6 s (Figure 9), the QBC strategy reaches at least the baseline performance with 1,512 instances (42%, out of the 3,599 total) for all classification models using the class-balancing algorithm. This represents a reduction of 58% in the training data used to achieve at least a similar performance. Moreover, a distinction is noticed between the QBC strategy with and without the balancement technique in its initial cycles, as the class-balancement algorithm shows considerable improvements compared to the standard QBC strategy at most steps evaluated in this experiment. Finally, it is essential to point out that using QBC, a window of 6 s, and the balancing strategy surpasses all baseline models’ performances for this setup.

### 3.4 EMC strategy on 3 and 6 s windows

**FIGURE 10:**
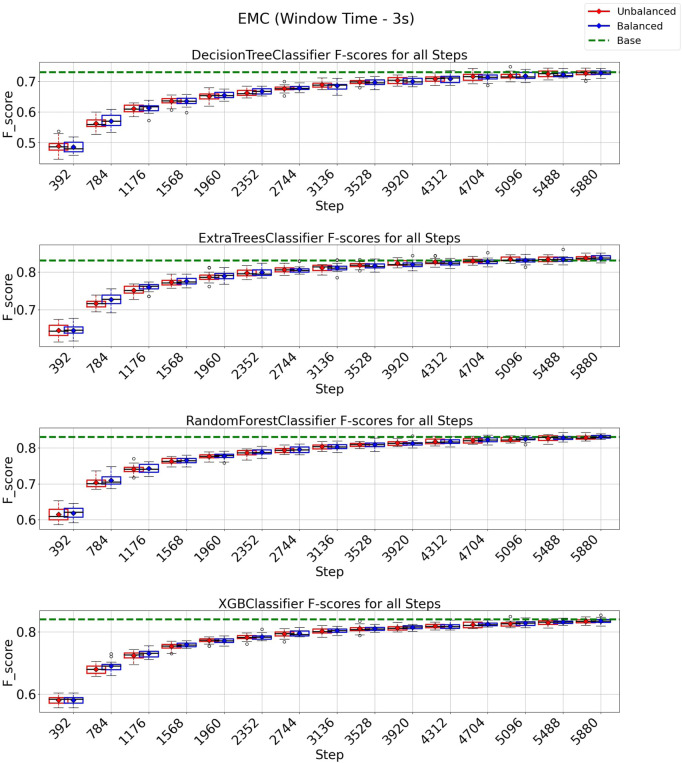
F1-score plots of the expected model change strategy for the 3-s window.

**FIGURE 11:**
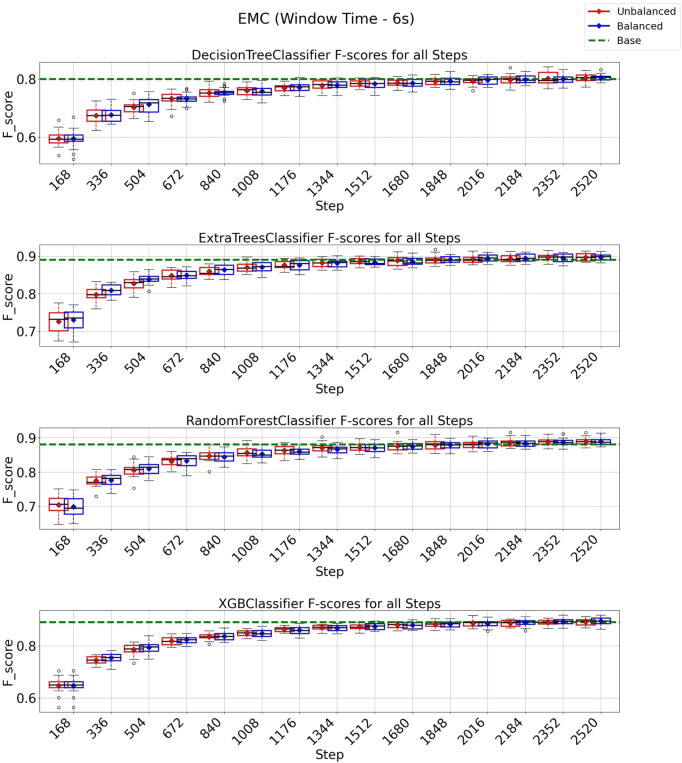
F1-score plots of the expected model change strategy for the 6-s window.

In the case of the EMC strategy, all classification models reach at least their baseline score for a window of 3 s, as shown in Figure 10 with a budget of 5880 instances from a total of 8,399 instances (70% of the entire data set). It is essential to observe from the experiments depicted in Figure 10 that the class-balancing algorithm indeed increased the performance of the EMC strategy compared to the standard EMC implementation, mainly in the early cycles of the EMC strategy. For a window of 6 s (Figure 11), the EMC strategy reaches at least the baseline performance with 2,016 instances (56%, out of the 3,599 total) for all classification models using the class-balancing algorithm. This represents a reduction of 44% in the training data used to achieve at least a similar performance. Similarly to the QBC strategy, a distinction is noticed between the EMC strategy with and without the balancement technique in its initial cycles, as the class-balancement algorithm shows improvements compared to the standard EMC strategy at most steps evaluated in this experiment. Finally, it is essential to point out that using EMC, a window of 6 s, and the balancing strategy surpasses all baseline models’ performances for this setup.

### 3.5 Comparing window sizes and active learning strategies performances

In this experiment, first, we evaluate the statistical significance of the difference between the window sizes (3 and 6 s) per model and AL strategy. Here, the objective is to ensure that the differences are statistically significant to affirm that one window size is better than the other for our problem. Therefore, we created Table 4 as follows. We used all experiments with the class-balanced strategy for both UNC and QBC, for all models and window sizes with the maximal budgets of 5880 (represents a total of 70% of the processed data using our pipeline) for 3 s windows and 2,520 (represents a total of 70% of the processed data using our pipeline) for 6 s windows. These budget values were chosen to ensure all combinations of AL strategies and machine-learning models could have enough data to at least reach the baseline performance and, therefore, have a more fair comparison between all combinations. We averaged all these experiments and also reported the standard deviations.

**TABLE 4:** f1-score of AL strategies for different windows and models.

Models	UNC		QBC		EMC	
	Window size		Window size		Window size	
	3 s	6 s	3 s	6 s	3 s	6 s
Decision Tree	72.60 ± 1.26	80.67 ± 1.39	72.94 ± 1.00	81.12 ± 1.34	72.81 ± 0.98	80.52 ± 1.14
Extra Tree	84.24 ± 0.71	**90.25** ± **0.92**	84.21 ± 0.58	90.13 ± 1.03	83.72 ± 0.78	89.71 ± 0.95
Random Forest	83.53 ± 0.79	89.13 ± 1.24	83.44 ± 0.66	89.15 ± 1.09	83.10 ± 0.48	88.86 ± 1.13
XGBoost	83.76 ± 0.86	89.80 ± 1.08	84.02 ± 0.87	89.58 ± 1.46	83.52 ± 0.81	89.48 ± 1.41

As seen in Table 4, using a window of 6 s always surpasses a window of 3 s regardless of the machine learning models and AL strategy adopted, with average f1-score differences at most 8%. We conducted a Wilcoxon signed rank test between each model’s average for a 3 and 6-s window and per AL strategy first. We verified that all these averages are indeed statistically significantly different for the window sizes of 3 and 6 s per AL strategy as the *p*-values were all below the threshold (i.e., with *p* − *value* < 0.05, we reject the null hypothesis that those averages come from the same distribution). Therefore, we can affirm that using a window size of 6 s is the best choice for our problem for all AL strategies.

Using Table 4, we also verified which combination of a machine learning model, 6-s window, and AL strategy (using the balanced strategy) would perform best in our problem. As seen in Table 4, an Extra Tree trained with Uncertainty Sampling and a 6-s window reached a 90.25% f1-score, and therefore, it is the best result achieved. We executed a Wilcoxon signed rank again, comparing this best average value with all other combinations of the 6-s window and AL strategy (using the balancing strategy), and the statistical values showed that this choice was better than all others. Therefore, we conclude that Extra Tree trained with Uncertainty Sampling and the class-balance technique are our problem’s best machine-learning model choices for our tactile sensing classification problem for a total budget of 70% of the training data given to our entire data pipeline.

## 4 Discussion

In this paper, we tested and improved three standard AL strategies for a texture classification task. To the best of our knowledge, this is the first work on using AL for tactile texture classification. Although we used the same sensor and experimental process as in Lima et al. (2021), we worked on some limitations of their methodology. The authors in Lima et al. (2021) had to undergo an expensive training process as they used 12 s of data exploration and trained their model on such a massive amount of data. Despite this significant effort, the training approach seemed to lack attention to the temporal features that could play a crucial role in the final outcome as they used each value from the sensor as a feature.

Our work advances existing texture classification methodologies by proposing a novel pipeline for tactile data used for texture classification. This pipeline includes a time window approach for extracting features and using AL with a class balancing algorithm. Therefore, we addressed a significant issue in tactile data for texture classification: training a high-performance machine learning model with a limited number of training data. Building such a high-performance machine learning model has several implications, including the possibility of deployment of low-complexity models in low-cost robotic hardware and the time reduction for the robotic arm exploration for classifying textures using tactile sensors. Our proposed pipeline achieved competitive and even better classification performance than an established baseline, with less annotated data, making the learning process faster and more efficient. We show that using at most 70% of the data available is enough to achieve and surpass the baseline performances using our proposed pipeline and algorithms. Moreover, we considered the effect of temporal features using our sliding overlapping windows and extracting the sensors’ data distributions. This gave the machine learning models detailed information regarding the robotic exploration, improving the performance of machine learning models learned from the tactile data. The AL strategies applied to the processed data further reduced the need for labeling training instances, as they selected only the most valuable ones to be labeled and given to the classifiers. As observed in the plots for 6-s windows, the learning process achieved the baseline results faster (with a lower budget for instances selected) and more efficiently (achieving an average f1-score of 90.21%) compared to 3-s windows. We believe that such a result was because a window of 6 s in the conducted experiments ensures that the robot explores the texture in the two axes. Therefore, changes in texture in both axes could be better characterized by the extracted features. The results also show that in some of our 3-second-window experiments, the baseline method outperforms specific active learning configurations, specifically the Decision Tree with uncertainty sampling and all Query by Committee (QBC) combinations. We believe this can be attributed to several key factors. Firstly, the discrepancy in performance between the baseline and active learning for the 3-s window experiments can be linked to the unique characteristics of our dataset and the duration of tactile exploration. Our dataset is tailored to focus on textures that primarily exhibit variations along the *x* and *y*-axes. A 3-s window, however, typically encompasses only one dimension of exploration, resulting in limited diversity in the captured data. Consequently, when the entire labeled dataset is available for model training, simpler models like the Decision Tree may not perform better due to the absence of all data points. To optimize labeling efforts, we imposed a maximal budget of 70% for active learning, beyond which further labeling would yield diminishing returns. In contrast, the more complex models employed in active learning demonstrate superior performance with a lower labeling budget. Moreover, the specific approach of bagging with randomly selected elements employed in QBC for creating the voting committee may inadvertently limit diversity in the committee models, affecting the method’s performance in 3-second-window experiments. In contrast, our 6-s window experiments demonstrate a different pattern, with active learning methods consistently outperforming the baseline. This can be attributed to the extended exploration duration covering at least two texture dimensions. This aligns more closely with the inherent characteristics of our dataset and showcases the efficacy of active learning strategies in scenarios where exploration durations offer a more comprehensive view of the tactile data’s complexity.

Furthermore, our balancement algorithm makes our AL strategies robust to the distribution imbalance from queries performed by standard AL strategies. The balancing algorithm showed a marginal effect on the UNC strategy, but it improved the results for the QBC and EMC strategies, mainly in the early stages of our process. Finally, combining a standard UNC strategy with the balancing algorithm and an ExtraTree as the machine learning model produced the best result for our tactile texture classification dataset. In conclusion, this work made the process of texture classification using tactile sensors more precise and efficient for real-world unstructured and dynamic environments by surpassing the results obtained by previous works.

We intend to expand this work in several directions. First, we would like to test and adapt our proposed pipeline for different types of tactile sensors and other sensory data. This approach would involve understanding how it performs with varying factors associated with different sensors. Extending this work to other dynamic real-world environments, such as industrial ones, would provide insights into the robustness of the pipeline. This work mainly focused on UNC, QBC, and EMC as active learning strategies. Future research endeavors include exploring alternative Active Learning (AL) strategies to assess their potential advantages and insights within tactile texture classification. Another path we intend to explore is how AL would perform in scenarios with incomplete data, such as those caused by partial contact between the tactile sensor and the examined object. We believe AL has the potential to select those instances that a model deems more uncertain and, therefore, learn from them and adapt over time, gradually becoming proficient in handling such scenarios.

## Data Availability

Publicly available datasets were analyzed in this study. This data can be found here: https://data.mendeley.com/datasets/n666tk4mw9/1.
